# Toward an Extended Definition of Major Depressive Disorder Symptomatology: Digital Assessment and Cross-validation Study

**DOI:** 10.2196/27908

**Published:** 2021-10-28

**Authors:** Nayra A Martin-Key, Dan-Mircea Mirea, Tony Olmert, Jason Cooper, Sung Yeon Sarah Han, Giles Barton-Owen, Lynn Farrag, Emily Bell, Pawel Eljasz, Daniel Cowell, Jakub Tomasik, Sabine Bahn

**Affiliations:** 1 Cambridge Centre for Neuropsychiatric Research Department of Chemical Engineering and Biotechnology University of Cambridge Cambridge United Kingdom; 2 Princeton Neuroscience Institute Princeton University Princeton, NJ United States; 3 UC San Diego School of Medicine University of California San Diego, CA United States; 4 Owlstone Medical Ltd Cambridge United Kingdom; 5 Psyomics Ltd Cambridge United Kingdom; 6 Sentinel Oncology Ltd Cambridge United Kingdom

**Keywords:** major depressive disorder, subthreshold depression, transdiagnostic symptoms, digital assessment, digital mental health, mobile phone

## Abstract

**Background:**

Diagnosing major depressive disorder (MDD) is challenging, with diagnostic manuals failing to capture the wide range of clinical symptoms that are endorsed by individuals with this condition.

**Objective:**

This study aims to provide evidence for an extended definition of MDD symptomatology.

**Methods:**

Symptom data were collected via a digital assessment developed for a delta study. Random forest classification with nested cross-validation was used to distinguish between individuals with MDD and those with subthreshold symptomatology of the disorder using disorder-specific symptoms and transdiagnostic symptoms. The diagnostic performance of the Patient Health Questionnaire–9 was also examined.

**Results:**

A depression-specific model demonstrated good predictive performance when distinguishing between individuals with MDD (n=64) and those with subthreshold depression (n=140) (area under the receiver operating characteristic curve=0.89; sensitivity=82.4%; specificity=81.3%; accuracy=81.6%). The inclusion of transdiagnostic symptoms of psychopathology, including symptoms of depression, generalized anxiety disorder, insomnia, emotional instability, and panic disorder, significantly improved the model performance (area under the receiver operating characteristic curve=0.95; sensitivity=86.5%; specificity=90.8%; accuracy=89.5%). The Patient Health Questionnaire–9 was excellent at identifying MDD but overdiagnosed the condition (sensitivity=92.2%; specificity=54.3%; accuracy=66.2%).

**Conclusions:**

Our findings are in line with the notion that current diagnostic practices may present an overly narrow conception of mental health. Furthermore, our study provides proof-of-concept support for the clinical utility of a digital assessment to inform clinical decision-making in the evaluation of MDD.

## Introduction

### Background

Major depressive disorder (MDD) is a common and heterogeneous condition representing the leading cause of disability worldwide [1]. MDD has been associated with poor global outcomes, including impaired social functioning, lower quality of life, inability to return to work, and suicide [2]. The condition is typically diagnosed in primary care settings, with most help seekers exhibiting subthreshold or subsyndromal presentations of the disorder [3,4]. Critically, recognizing diagnosable symptomatology of MDD can be particularly challenging, with any 2 individuals’ meeting criteria for the condition potentially having no symptoms in common [5]. In fact, short consultation times coupled with the complexity and subjectivity of diagnosing MDD results in primary care practitioners misdiagnosing >50% of low-mood help seekers [6]. This means that many patients do not receive the most effective treatment and support.

In an attempt to improve the current diagnostic practice, the search for objective diagnostic tests and valid biomarkers for depression has received a lot of attention. However, despite substantial research expenditures and large-scale genome-wide studies, no pathognomonic biological markers of depression have been identified [7-11]. In fact, with the exception of a few neuropsychiatric disorders, not a single psychiatric diagnosis can still be validated by molecular, genetic, or imaging biomarkers [12]. Importantly, psychiatric diagnostic criteria were not conceptualized to facilitate biological differentiation [13], with extensive comorbidity across conditions being the rule rather than the exception [14] and no single condition representing a discrete entity [15-17].

Another issue pertaining to psychiatric nosology is *incomplete symptom capture* [18,19]. It has been argued that the symptom profiles described in diagnostic manuals, such as the *Diagnostic and Statistical Manual of Mental Disorders*, *Fifth Edition* (*DSM-5*) [20] and the *International Statistical Classification of Diseases*, *Eleventh Revision* (*ICD-11*) [21], may be overly narrow, failing to capture the wide range of clinical symptoms that are endorsed by individuals with MDD [18]. For instance, although anxiety is not listed as a core symptom of the condition, many individuals with MDD experience co-occurring symptoms of anxiety and typically meet the criteria for at least one anxiety disorder [22-26].

Some authors suggest that assessing the presence of anxiety symptoms in patients with MDD is critical [27,28]. Others propose combining depression and anxiety disorders, which present with largely overlapping symptomatology. Combining these disorders may be a useful strategy for better clinical evaluation and management of patients with MDD [29]. This is important, given that estimates of prevalence rates for treatment-resistant depression range between 30% and 50% [30,31], with incomplete remission often leading to relapse [32], increased chronicity and severity of episodes [33], greater functional impairments [34], and higher risk of suicide [35]. One of the established risk factors that predispose patients to develop treatment-resistant depression are comorbid anxiety symptoms or anxiety disorders, especially generalized anxiety disorder (GAD) [31].

In this regard, a transdiagnostic view of MDD encompassing symptoms of anxiety and other commonly co-occurring disorders may improve early and accurate diagnosis, reflect biological disease understanding (eg, twin studies have shown shared genetic predisposition for MDD and GAD [36]), and allow for personalized treatment strategies. An extended definition of MDD symptomatology may also reduce the misdiagnosis of bipolar disorder (BD) as MDD [37,38], which is particularly problematic, with many individuals having to wait 8-10 years before receiving a correct diagnosis [39,40].

Critically, time is premium in primary care settings, where relying on brief symptom-count checklists, such as the Patient Health Questionnaire–9 (PHQ-9) [41], is a common practice. Importantly, the PHQ-9 may overestimate depression severity in primary care patients relative to other self- and clinician-rated scales [42], resulting in a greater reliance on medication as a first-line treatment option and an increased potential for adverse drug effects [43,44]. In addition, some researchers have suggested that the PHQ-9 may be missing the presence of symptoms that are meaningful for patients and that longer assessments may be better at capturing diagnosable levels of low mood [45]. To this end, digital technologies allow for the cost- and time-effective collection of a vast range of important patient and symptom data [46]. Such an approach offers an innovative way to improve and advance mental health care provision. In turn, the use of digital technologies could help alleviate the load on the health care system by providing individuals with subthreshold or mild MDD with self-help tips and psychoeducation. This would reserve the limited and specialized services for more severe or highly comorbid and complex patients.

### Objectives of This Study

This study aims to provide evidence for an extended definition of MDD symptomatology using a digital assessment that was developed for the delta study [47]. The digital assessment was designed following an extensive analysis of existing validated questionnaires for mood disorders [48-57], the *DSM-5* [20], and the *International Statistical Classification of Diseases, Tenth Revision* (*ICD-10*) [21], as well as input from psychiatrists and a service user group. In an attempt to move away from a symptom-count approach to psychopathology, our study uses machine learning (ML) methods (advanced statistical and probabilistic techniques that automatically learn from data) to construct a data-driven view of MDD. We examined the extent to which (1) disorder-specific symptoms (ie, symptoms of depression) and (2) transdiagnostic symptoms (ie, cross-disorder symptoms) could be used to answer the following question: “when does depression become a mental disorder?” To do this, we compared individuals with MDD with those with subthreshold levels of depressive symptoms. Although this was largely an exploratory study, it was predicted that, relative to a disorder-specific model of psychopathology, an extended model would be better at identifying individuals with diagnosable levels of MDD. In particular, we predicted that symptoms of anxiety would be highly indicative of the disorder.

## Methods

### The Delta Study

This study used data from the delta study that was conducted by the Cambridge Centre for Neuropsychiatric Research between April 2018 and November 2019. Olmert et al [47] provided a detailed description of the delta study design and sampling procedures. In brief, the key objectives of the study were to develop and validate a diagnostic algorithm to (1) reduce the misdiagnosis of MDD and BD and (2) achieve a more accurate and earlier diagnosis of MDD in individuals presenting with depressive symptoms. The target population for the primary objective was those who had received a recent diagnosis of MDD (within the past 5 years) by a general practitioner or psychiatrist and those who were experiencing depressive symptoms at the time of recruitment. The target population for the secondary objective included those without a previous mood disorder diagnosis and who were experiencing depressive symptoms at the time of recruitment. Individuals aged between 18 and 45 years could take part in this study. This age group was selected in consultation with a practicing psychiatrist (SB) on the basis that individuals aged between 18 and 45 years are most likely to have undiagnosed BD (primary objective of the delta study). Further inclusion criteria were being a resident in the United Kingdom, not pregnant or breastfeeding, not suicidal, and a score of at least five on the PHQ-9 [41]. Information on treatment history was collected but was not deemed an inclusion or exclusion criterion. All participants provided informed consent to participate in the study, which was approved by the University of Cambridge Human Biology Research Ethics Committee (approval number HBREC 2017.11).

Over 5000 participants were recruited on the web through email, via paid Facebook (Facebook Inc) advertisements, and updates on the Cambridge Centre for Neuropsychiatric Research laboratory website. Eligible participants were invited to take part in the main study; of these, 3232 completed the digital assessment via the delta study website. The digital assessment was designed following an extensive analysis of validated questionnaires for mood disorders [48-57], the *DSM-5* [20], and the *ICD-10* [21], as well as input from psychiatrists to ensure the inclusion of a wide range of clinically meaningful and well-validated symptoms of MDD and BD and other symptoms of interest (eg, other psychiatric conditions). The assessment was further refined following advice from a service user group on features, including tone of voice and user journey. For each participant, an individualized dashboard guided their progress through the study. The digital assessment could be completed on a smartphone, laptop, tablet, or desktop computer and comprised 635 distinct questions. One question was presented at a time, with participants required to select the answer that best described their feelings and experiences (eg, from “No, not at all” to “Yes, very much”). The questions were grouped into six sections: (1) demographics and personal history; (2) bipolar and manic and hypomanic symptoms; (3) depressive symptoms; (4) personality traits; (5) medication, treatment, and substance use; and (6) other psychiatric disorders and symptoms, including GAD, social anxiety disorder, emotional instability, panic disorder, eating disorders, insomnia, and obsessive-compulsive disorder. Participants were required to press *next* to take them to the following question. Each section could be completed in 10-15 minutes, although response times varied because of the adaptive nature of the assessment, where only relevant questions were asked based on responses to previous questions. 

A subgroup of the original study cohort (n=1740) consented to provide dried blood spot samples and complete a telephone interview for MDD and BD using the *Diagnostic and Statistical Manual of Mental Disorders*, *Fourth Edition* (*DSM-IV*)–based Composite International Diagnostic Interview (CIDI [48]), with 924 participants completing both steps. Of these 924 participants, 241 (26.1%) self-reported having been diagnosed with MDD by a general practitioner or psychiatrist and were confirmed to have met the criteria for the condition by the CIDI. Of these 241 participants, 64 (26.6%) participants (11/64, 17% participants were male) met the criteria for *current* MDD (ie, symptoms present in the past month according to the CIDI). This formed the MDD group. The subthreshold depression group included 15.2% (140/924) participants (male: 35/140, 25%) who self-reported no diagnosis of MDD and whose symptoms of depression were confirmed to not meet the criteria for MDD according to the CIDI. None of the selected participants had ever experienced a manic or hypomanic episode.

### Data Analytic Strategy

#### Participant Characteristics

Participant characteristics and comorbidities were collected via digital assessments and are shown in Table 1. Group differences in continuous variables were explored using Mann–Whitney U tests as the data were nonnormally distributed, with effect sizes reported as *r* (small ≥0.1; medium ≥0.3; and large ≥0.5) [58]. Group differences in categorical variables were evaluated using chi-square tests or Fisher exact test for low-frequency data (ie, values <5). Effect sizes are reported as Cramer V (*φ_c_*; small ≥0.1; medium ≥0.3; and large ≥0.5) [58].

**Table 1:** Participant characteristics and comorbidities: major depressive disorder versus subthreshold depression group comparisons.

Characteristics			Subthreshold depression (n=140)		MDD^a^ (n=64)		*U* ^b^		*P* value		*r* ^c^		Chi-square (*df*)		*φ*_c_^d^
Age (years), mean (SD)			25.84 (6.66)		25.94 (5.7)		4209		.49		0.05		N/A^e^		N/A
BMI, mean (SD)			24.62 (4.73)		28.29 (6.8)		2901		<.001		0.28		N/A		N/A
**Sex, n (%)**															
	Male	49 (35)		11 (17.2)		N/A		N/A		N/A		N/A		N/A	
	Female	91 (65)		53 (82.8)		N/A		.01		N/A		6.7 (1)		0.18	
**Higher education^f^, n (%)**															
	Yes	87 (62.1)		37 (42.2)		N/A		N/A		N/A		N/A		N/A	
	No	53 (37.1)		27 (57.8)		N/A		.56		N/A		0.4 (1)		0.04	
**Employment, n (%)**															
	Employed	81 (57.9)		35 (54)		N/A		N/A		N/A		N/A		N/A	
	Unemployed	5 (3.6)		8 (12.5)		N/A		N/A		N/A		N/A		N/A	
	Student	54 (38.6)		21 (32.8)		N/A		.05		N/A		6.0 (2)		0.17	
**Support network: relationships, n (%)**															
	Secure and stable relationship	84 (59.6)		133 (55.4)		N/A		N/A		N/A		N/A		N/A	
	Insecure and unstable relationship	9 (6.4)		14 (5.8)		N/A		N/A		N/A		N/A		N/A	
	Single	48 (34)		93 (38.8)		N/A		.54		N/A		1.3 (2)		0.08	
**Support network: living alone, n (%)**															
	Yes	9 (6.4)		9 (14.1)		N/A		N/A		N/A		N/A		N/A	
	No	131 (93.6)		55 (85.9)		N/A		.07		N/A		3.2 (1)		0.13	
**Psychiatric history, n (%)**															
	GAD^g^	12 (8.6)		47 (73.4)		N/A		<.001		N/A		89.9		0.66	
	Personality disorder	0		3 (4.7)		N/A		N/A		N/A		N/A		N/A	
	OCD^h^	3 (2.1)		3 (4.7)		N/A		.28		N/A		0.4 (2)		0.07	
	Panic disorder	0		7 (10.9)		N/A		N/A		N/A		N/A		N/A	
	Social anxiety	1 (0.7)		9 (14.1)		N/A		<.001		N/A		16.8 (2)		0.29	
	Eating disorder	2 (1.4)		5 (7.8)		N/A		.03		N/A		5.4 (2)		0.16	
**Medical history, n (%)**															
	Thyroid disease	4 (2.9)		2 (3.1)		N/A		.99		N/A		0.0 (3)		0.01	
	Cardiovascular disease	1 (0.7)		0		N/A		N/A		N/A		N/A		N/A	
	Irritable bowel syndrome	6 (4.3)		2 (3.1)		N/A		.99		N/A		0.2 (3)		0.03	
	Chronic pain	30 (21.4)		14 (21.9)		N/A		.94		N/A		0.0 (3)		0.01	
	Migraines	53 (37.9)		31 (48.4)		N/A		.15		N/A		2.0 (3)		0.10	
**Current psychiatric treatment, n (%)**															
	SSRI^i^ antidepressants	12 (8.6)		32 (50)		N/A		<.001		N/A		44.6 (3)		0.47	
	SNRI^j^ antidepressants	0		5 (7.8)		N/A		N/A		N/A		N/A		N/A	
	Tricyclic antidepressants	0		3 (4.7)		N/A		N/A		N/A		N/A		N/A	
	Other antidepressants	1 (0.7)		8 (12.5)		N/A		<.001		N/A		14.5 (3)		0.27	
	Anxiety medication	4 (2.9)		10 (15.6)		N/A		.002		N/A		11.2 (3)		0.23	
	Antipsychotics	0		3 (4.7)		N/A		N/A		N/A		N/A		N/A	
	Mood stabilizers	0		2 (3.1)		N/A		N/A		N/A		N/A		N/A	
	Psychotherapy	4 (2.9)		16 (25)		N/A		<.001		N/A		24.4 (3)		0.35	

^a^MDD: major depressive disorder.

^b^Mann–Whitney U test.

^c^Effect size (*r*).

^d^Effect size (Cramer V).

^e^N/A: not applicable.

^f^Undergraduate degree or equivalent and above was coded as *yes*, whereas A level or equivalent and below was coded as *no*.

^g^GAD: generalized anxiety disorder.

^h^OCD: obsessive-compulsive disorder.

^i^SNRI: serotonin–norepinephrine reuptake inhibitor.

^j^SSRI: selective serotonin reuptake inhibitor.

#### Model Construction and Performance

Random forest classification models were constructed in Python 3.7.4 (Python Software Foundation) using the scikit-learn library 0.21.3 to distinguish between MDD and subthreshold depression using (1) disorder-specific symptoms (ie, symptoms of depression), and (2) transdiagnostic symptoms (ie, cross-disorder symptoms). We constructed two models: a *depression model*, including 36 symptoms of depression and an *extended model*, comprising 134 symptoms (36 symptoms of depression, 12 symptoms of GAD, 19 symptoms of BD or mania, 15 symptoms of hypomania, 6 symptoms of social anxiety, 11 symptoms of emotional instability, 14 symptoms of panic disorder, nine symptoms of obsessive-compulsive disorder, two symptoms of eating disorders, and 10 symptoms of insomnia). These symptoms were manually selected based on the maximum number of available symptoms from the digital assessment. Scores per symptom ranged from 0-1, with higher scores indicating increased severity. Mean symptom severity per group can be found in Table S1, Multimedia Appendix 1.

Although some symptoms overlapped across disorders (eg, tiredness, low energy, and irritability), we did not feel that it would be appropriate to combine these as the questions were framed in the context of each condition. Furthermore, although all participants were asked about symptoms of depression, BD or mania, and hypomania, the questions for the remaining conditions were adaptive in nature, such that only relevant questions were asked based on responses to previous questions. This resulted in some participants having *missing* data. These data were imputed as zeros. Furthermore, owing to the adaptive nature of the digital mental health assessment, participants answered questions on current (eg, present in the past 2 weeks) or past symptoms of all disorders.

For each of the models, nested cross-validation (NCV) was performed to obtain the highest algorithmic accuracy while ensuring the generalizability of the models. At each iteration of NCV, the data were randomly split into three folds; two-thirds of the data were used in the inner loop for model training and validation, and one-third was used for testing the model in the outer loop. The inner loop was (further) randomly split into three folds, whereby the hyperparameters (ie, number of estimators and maximum depth) were tuned, and the best cross-validated model was selected. To do this, two of the three folds were used to tune the model parameters and train the model, which was then validated on the third fold. This procedure was repeated with the remaining combinations of training and validation folds. The final model (ie, the optimized classifier) was obtained by fitting a model with the tuned parameters to all three data folds from the inner loop and then evaluating the hold-out test data in the outer loop. This procedure was repeated 100 times with different splits of the data (into train and test sets), resulting in a total of 300 unique models for each feature set (ie, depression model vs extended model).

Model performance was evaluated by measuring the area under the receiver operating characteristic curve (AUC) for the 300 models and averaging across all models for each feature set. The AUC shows the degree of separability between two conditions (ie, MDD vs subthreshold depression) and represents the probability that a randomly selected subject with the condition is rated or ranked as more likely to have the condition than a randomly selected individual without the condition (AUC: ≥0.9=excellent; ≥0.8=good; ≥0.7=fair; ≥0.6=poor; ≥0.5=fail) [59]. Mann–Whitney U tests were used to determine significant differences in AUCs across the 300 models between the depression and transdiagnostic models.

The mean sensitivity, specificity, and accuracy scores per model were also evaluated. Here, sensitivity refers to the model’s ability to classify MDD cases correctly (ie, true positives), whereas specificity refers to the model’s ability to classify subthreshold depression cases correctly (ie, true negatives). Accuracy corresponds to the model’s ability to classify all true cases (ie, both true positives and true negatives).

#### Feature Importance and Occurrence

Relative feature importances (ie, Gini impurity [60]) were calculated for the 300 models and averaged across all models for each feature set, with features with higher values showing better discrimination between MDD and subthreshold depression. Feature occurrence was calculated by summing the number of times each feature contributed to each of the 300 models and computing a percentage score per feature for each feature set, with higher values representing higher feature occurrence.

#### Diagnostic Performance of the PHQ-9

Finally, to establish the diagnostic performance of the PHQ-9 on the basis of its intended use, we calculated the sensitivity, specificity, and accuracy in the current sample using the standard cut-off score of ≥10 [41]. This mimics what would ordinarily happen in the clinic (ie, those scoring ≥10 would be classified as MDD).

## Results

### Participant Characteristics

Table 1 presents information on the characteristics of each group, with statistical comparisons. The groups did not differ significantly in age, level of education, support network (ie, relationships and living conditions), or medical history. However, the MDD group had a significantly higher proportion of women and a higher mean BMI than the subthreshold depression group. The MDD group was significantly more likely to be unemployed than the subthreshold depression group, which was more likely to be employed or in full-time education. The groups had different psychiatric histories, with the MDD group having a significantly higher proportion of individuals with comorbid GAD, social anxiety disorder, and eating disorders. Finally, relative to the subthreshold depression group, individuals with MDD were more likely to be currently taking psychiatric medication and receiving psychotherapy.

### Depression Model

**Figure 1:**
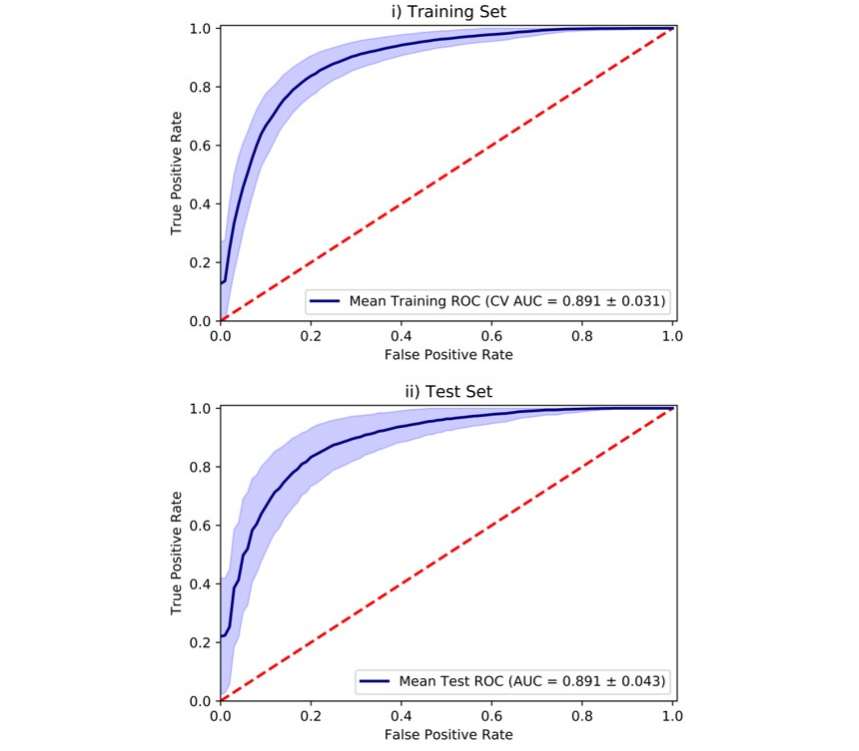
Area under the receiver operating characteristic curves showing mean predictive performance of the depression model. The models were applied to predict the probability of major depressive disorder in the: (1) training and (2) test sets. AUC: area under the receiver operating characteristic curve; CV AUC: cross-validated area under the receiver operating characteristic curve; MDD: major depressive disorder; ROC: receiver operating characteristic.

This model comprised 36 features, with analyses demonstrating good discriminatory performance on both the training (AUC=0.89±0.03) and test sets (AUC=0.89±0.04; Figure 1). Approximately 81% (52/64) of the MDD cases and 81.4% (114/140) of the subthreshold depression cases were correctly classified by the model, corresponding to the mean sensitivity and specificity scores, respectively. The mean accuracy of the model was 81.6%, corresponding to the proportion of individuals correctly classified by the model.

**Figure 2:**
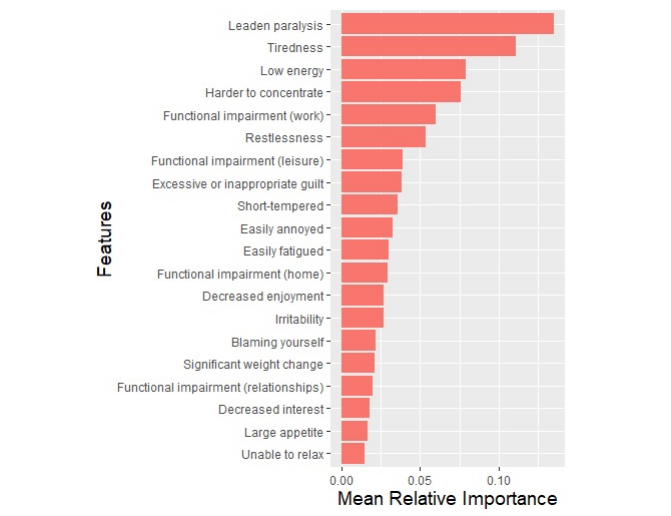
Top 20 mean relative importance for the depression-specific model. Features have been ordered from most to least important.

The top 20 features contributing to the depression model (averaged across all 300 models) were leaden paralysis, tiredness, low energy, harder to concentrate, functional impairment (work), restlessness, functional impairment (leisure), excessive or inappropriate guilt, short-tempered, easily annoyed, easily fatigued, functional impairment (home), decreased enjoyment, irritability, blaming oneself, significant weight change, functional impairment (relationships), decreased interest, large appetite, and unable to relax. Figure 2 shows the mean relative feature importances. These features appeared across at least 81.3% (244/300) of the models. Multimedia Appendices 2 and 3 show the relative feature importances and percentage occurrences of all 36 features.

### Extended Model

Next, we added 98 features to the model, resulting in an extended model comprising 134 features. Analyses demonstrated excellent discriminatory performance on both the training (AUC=0.94±0.03) and test sets (AUC=0.94±0.04; Multimedia Appendix 4). Mann–Whitney U tests confirmed a significant improvement in model performance (ie, AUC) relative to the depression-specific model (training set: *U*=12922.50, *P*<.001; test set: *U*=33525.50, *P*<.001). Here, 83% (53/64) of MDD cases (ie, sensitivity) and 90% (126/140) of subthreshold depression cases (ie, specificity) were correctly classified by the model, whereas the ability of the model to correctly classify both MDD and subthreshold depression cases was 87.7% (ie, accuracy). Feature importances can be found in Multimedia Appendix 5, with percentage occurrences found in Multimedia Appendix 6.

On the basis of these findings, we then reran the analyses using a *truncated* version of the extended model, which only included features that appeared across at least 90.3% (271/300) of the models (Multimedia Appendix 6). The truncated model comprised 12 symptoms of depression, 11 symptoms of GAD, six symptoms of insomnia, three symptoms indicative of emotional instability, and one panic disorder symptom, resulting in a total of 33 features.

**Figure 3:**
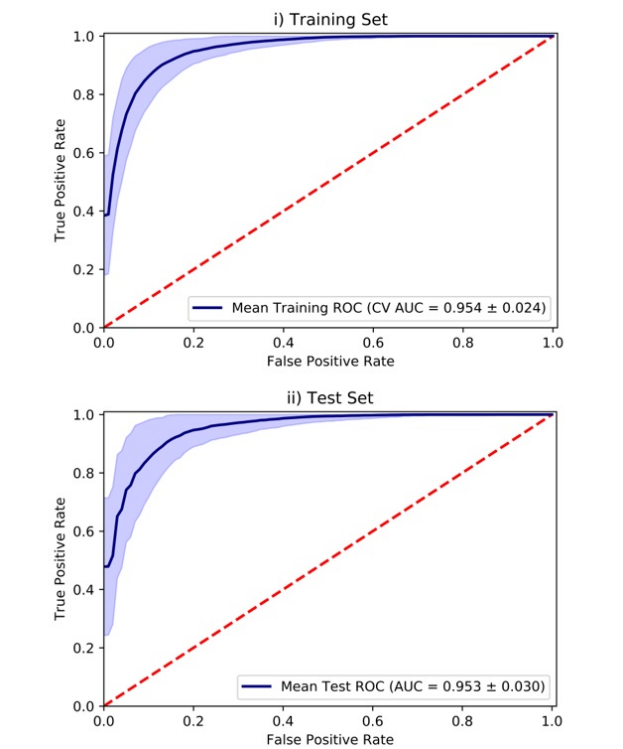
Area under the receiver operating characteristic curves showing mean model performance of the truncated version of the extended model. The models were applied to predict the probability of major depressive disorder in the: (1) training and (2) test sets. AUC: area under the receiver operating characteristic curve; CV AUC: cross-validated area under the receiver operating characteristic curve; MDD: major depressive disorder; ROC: receiver operating characteristic.

**Figure 4:**
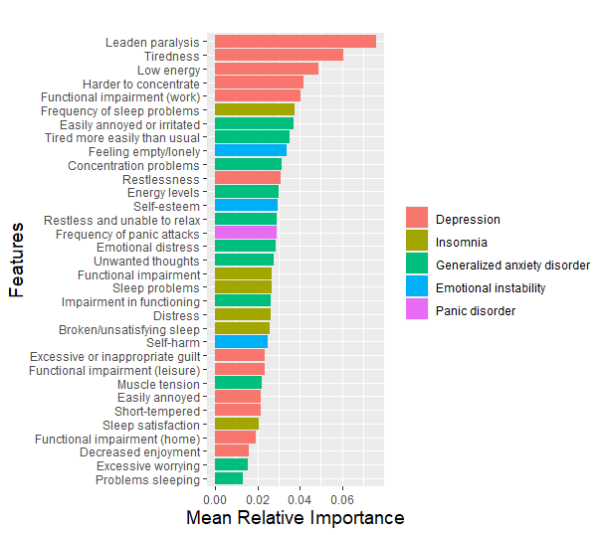
Mean relative importance for the 33 features in the truncated version of the extended model. Features have been ordered from most to least important and colored according to the disorder or symptom cluster they correspond to.

The analyses revealed a significant improvement in the model’s discriminatory performance on both the training (AUC=0.95±0.02) and test sets (AUC=0.95±0.03; Figure 3) relative to the full extended model (training set: *U*=12856, *P*<.001; test set: *U*=322798.50, *P*<.001). The mean sensitivity, specificity, and accuracy scores were 86.5%, 90.8%, and 89.5%, respectively. The mean relative feature importance for the 33 features can be found in Figure 4, with percentage occurrences found in Multimedia Appendix 7.

### Diagnostic Performance of the PHQ-9

The sensitivity of the PHQ-9 for detecting MDD was 92.2%, whereas the specificity was 54.3%, and the overall diagnostic accuracy was 66.2%.

## Discussion

### Principal Findings

This study provides evidence for an extended definition of MDD symptomatology and supports the use of a digital assessment as an aid to clinical decision-making in the identification of MDD. Relative to a disorder-specific model of MDD psychopathology, an extended model of symptomatology was better at distinguishing between individuals with MDD and those with subthreshold levels of the disorder. In particular, a truncated version of the model, comprising symptoms of depression, GAD, insomnia, emotional instability, and panic disorder, demonstrated excellent predictive performance (AUC=0.95; sensitivity=86.5%; specificity=90.8%; and accuracy=89.5%).

Critically, although the PHQ-9 was particularly good at detecting MDD in the current sample, it tended to overdiagnose MDD in subthreshold depression and, in turn, was associated with poor overall diagnostic performance. Overdiagnosis of MDD presents a significant problem and has the potential for antidepressant overprescription and adverse drug effects in individuals who may benefit from alternative treatment options [43], such as psychotherapy or psychoeducation. Furthermore, relying on a simple cut-off score does not allow for personalized treatment plans and strategies, potentially resulting in incomplete remission rates.

Overall, the findings from our models are in line with the notion that current diagnostic practices may present a narrow conception of mental health that does not allow for the wide range of clinical signs and symptoms that are endorsed by individuals with MDD. Across our models, the most predictive symptom of MDD was leaden paralysis, which refers to an extreme form of fatigue or heavy, leaden feelings in the arms and legs. This finding is in line with a recent study by Han et al [61], whose findings revealed that leaden paralysis was a robust and important predictor of first-onset MDD. Critically, although leaden paralysis is included in the *DSM-5* specifier for atypical depression, it is not deemed a core feature of the disorder [20]. In fact, even fatigue or loss of energy and tiredness are not considered essential symptoms according to the *DSM-5* [20]. Importantly, exhaustion, extreme tiredness, and loss of energy are typically seen in primary care settings and are often the predominant presenting complaint [62]. In fact, in a large European study comprising approximately 2000 depressed primary care patients across 6 countries, almost two-thirds of patients reported feeling tired [63]. These findings suggest that leaden paralysis and its lesser extreme variants (ie, tiredness and low energy) may be particularly important for the recognition of MDD in the primary care setting.

As expected, symptoms of GAD were among the most predictive features of MDD, with *overlapping* symptoms between GAD and depression (eg, tiredness, low energy, and irritability) being particularly indicative of the disorder. For instance, regarding the latter, irritability has been seen to occur in one-third to one-half of patients with MDD [64-66] and is associated with greater severity and chronicity, a history of suicide attempts, and reduced quality of life [64]. Other GAD-specific symptoms that were highly predictive of MDD included unwanted thoughts, excessive worrying, and emotional distress. Importantly, it could be argued that our model simply reflects the significantly higher rates of comorbidity in the MDD group relative to the subthreshold depression group, particularly with regard to GAD. Similarly, our model may capture higher levels of severity or a higher p factor in the MDD group [67] rather than important components of the condition.

Although our findings should be interpreted with caution, our view is that this should not detract from the importance of assessing for transdiagnostic symptoms of MDD, especially as these are likely to share common underlying pathophysiology and genetics [36,68]. Indeed, the evaluation of anxiety symptoms in the context of MDD is critical, particularly given the chronicity of the conditions, with research indicating that anxiety disorders may be a precursor to MDD [69]. Notably, combining clinical information with biological biomarkers, such as serum analytes, can be used to predict the development of future depressive episodes in individuals presenting with social anxiety [70] and panic disorder [71]. Identifying those who may be at a heightened risk for comorbid anxiety and depression is of clinical importance, particularly given that these individuals are likely to exhibit more pervasive and recurrent forms of illness, reduced remission rates, and increased suicidality [72-74].

Indeed, suicidality has particularly been associated with the co-occurrence of depression and panic disorder [75,76], with our findings indicating that the frequency of panic attacks was an important predictor of MDD. Importantly, comorbid panic disorder and MDD have been related to increased depression severity, an earlier age of onset, increased functional impairment, and a poorer clinical prognosis [77], suggesting that assessing for panic disorder may be important when diagnosing and treating MDD. Similarly, our findings indicate that a more in-depth evaluation of the symptoms associated with sleep problems or insomnia, including emotional distress caused by disordered sleep, warrants inclusion when diagnosing MDD. This is particularly important as sleep problems have been shown to reduce the efficacy of depression treatment [78].

Finally, symptoms of emotional instability or personality disorder, including feeling empty, low self-esteem, and self-harm, were also seen to be important when distinguishing between MDD and subthreshold depression. Feeling empty or chronic emptiness has been closely related to depression and suicidal ideation [79]. Interestingly, a recent qualitative study in adolescents with depression revealed that a partial or complete blunting of any emotion (negative or positive), with feelings of flatness, emptiness, and lack of emotions, was an important component of anhedonia [80]. It is interesting that self-esteem but not self-worth (a diagnostic criterion for MDD) was a predictor of the disorder. This finding suggests that wording may also have an important impact on individuals’ subjective evaluations of their symptoms, with the concept of self-esteem perhaps being easier to grasp than that of self-worth. Finally, although diagnostic descriptions of MDD symptomatology include suicidality as a criterion for the disorder, expanding this to include self-harm may facilitate its identification.

Taken together, our findings indicate that the current diagnostic criteria for MDD may fail to evaluate relevant clinical information that is important for the diagnosis and treatment of individuals with the disorder. Although time is a luxury in the primary care setting, our study supports the use of digital technologies as a means for obtaining a more comprehensive depiction of MDD symptomatology in a time-efficient manner. Notably, related research using the same digital mental health assessment has highlighted the utility of the tool in distinguishing individuals with MDD from those with BD [81]. Indeed, digital technologies have the potential to aid in the recognition of a wide range of psychiatric conditions, allowing for more time to be spent managing and treating symptoms. In turn, digital technologies can reduce the number of in-person appointments, alleviate health care professionals’ workload, and reduce the risk of burnout. The use of digital technologies also has the potential to reduce some of the barriers associated with disclosing mental health difficulties, such as discomfort as well as issues related to stigma and discrimination. Furthermore, research has demonstrated that patients are more likely to report severe symptoms on technology platforms than to a health care professional [82] and value the independence and empowerment that can be obtained from the use of a digital platform [83].

### Strengths and Limitations

To our knowledge, this is the first study to provide evidence for an extended definition of MDD symptomatology using a digital assessment. Furthermore, the digital assessment was designed following an extensive analysis of existing validated questionnaires for psychiatric disorders and diagnostic manuals, as well as input from psychiatrists and a service user group. In addition, as opposed to the use of healthy controls as a reference population against which patients are compared, our subthreshold depression group represented a clinically relevant reference group. Finally, the use of ML methods meant that patterns in data could be more readily and accurately identified, whereas our NCV approach allowed us to obtain high algorithmic accuracy while ensuring the generalizability of the models.

This study also had several limitations. First, as with any supervised ML approach to psychopathology, our analyses were limited by the *truthfulness* of the diagnostic labels (MDD vs subthreshold depression). Second, given the adaptive (nonlinear) nature of the question flow, missing data were imputed with zeros, which may have resulted in an overly artificial data set. This should be borne in mind when interpreting these findings. In addition, the MDD sample was small in size and primarily comprised women, reflecting the higher prevalence of MDD in women than in men [84] and the difficulties in recruiting males with MDD. Furthermore, given that suicidality was an exclusion criterion in this study, measures of suicidal thoughts, ideations, plans, or impulses do not appear in our list of important features of MDD. This is a key limitation of the study, as suicidality denotes an important component of the condition. Similarly, symptoms from other disorders that frequently co-occur with MDD, such as posttraumatic stress disorder, were not available for inclusion in our analyses, which may have allowed for a more comprehensive depiction of MDD symptomatology.

### Conclusions

In an attempt to answer the question “when does depression become a mental disorder?”, our study demonstrated that a data-driven view of MDD may improve our understanding of the condition. A more comprehensive conceptualization of the psychopathology of MDD, including symptoms of depression, GAD, insomnia, panic disorder, and emotional instability, may not only facilitate patient stratification but also allow for personalized treatment plans and strategies. Although further studies with larger sample sizes are required to replicate our findings, our study shines a positive light on the use of digital technologies as an innovative way to help develop and facilitate mental health care provision. In particular, digital technologies have the capacity to collect a vast range of key clinical information that may be important for the diagnosis and treatment of individuals with MDD.

## Appendix

Multimedia Appendix 1 Mean symptom severity per group was separated by disorder or symptom clusters.

Multimedia Appendix 2 Depression model: mean relative feature importance.

Multimedia Appendix 3 Depression model: percentage feature occurrences.

Multimedia Appendix 4 Receiver operating characteristic curves showing the mean predictive performance of the extended model. The models were applied to predict the probability of major depressive disorder in the training and test sets.

Multimedia Appendix 5 Extended model: mean relative feature importance colored by disorder or symptom clusters.

Multimedia Appendix 6 Extended model: percentage feature occurrences colored by disorder or symptom clusters.

Multimedia Appendix 7 Truncated model: percentage of feature occurrences colored by disorder or symptom clusters.

## Abbreviations

AUC: area under the receiver operating characteristic curve
BD: bipolar disorder
CIDI: Composite International Diagnostic Interview
DSM-IV: Diagnostic and Statistical Manual of Mental Disorders, Fourth Edition
DSM-5: Diagnostic and Statistical Manual of Mental Disorders, Fifth Edition
GAD: generalized anxiety disorder
ICD-10: International Statistical Classification of Diseases, Tenth Revision
ICD-11: International Statistical Classification of Diseases, Eleventh Revision
MDD: major depressive disorder
ML: machine learning
NCV: nested cross-validation
PHQ-9: Patient Health Questionnaire–9
