# Supplementary material for: Toward an Extended Definition of Major Depressive Disorder Symptomatology: Digital Assessment and Cross-validation Study
Source: JMIR Form Res. 2021 Oct 28;5(10):e27908. doi: 10.2196/27908 (PMC8587324; doi:10.2196/27908)
Supplement: Multimedia Appendix 1 [file formative_v5i10e27908_app1.docx]

***Multimedia Appendix 1*Table 1.** Mean symptom severity per group separated by disorder/symptom cluster

|  |  | **Subthreshold depression**  **(*n* = 140)** | **MDD (*n* = 64)** |
| --- | --- | --- | --- |
| **Depression** |  | ***M* (SD)** | |
|  | **Low energy** | 0.42 (0.27) | 0.70 (0.24) |
|  | **Struggle to fall asleep** | 0.62 (0.49) | 0.64 (0.48) |
|  | **Unsatisfying sleep** | 0.63 (0.48) | 0.69 (0.47) |
|  | **Waking up early** | 0.50 (0.50) | 0.55 (0.50) |
|  | **Sleeping too much** | 0.36 (0.48) | 0.48 (0.50) |
|  | **Functional impairment (home)** | 0.37 (0.30) | 0.59 (0.27) |
|  | **Easily annoyed** | 0.46 (0.28) | 0.65 (0.26) |
|  | **Small appetite** | 0.24 (0.31) | 0.24 (0.31) |
|  | **Large appetite** | 0.16 (0.28) | 0.32 (0.37) |
|  | **Low self-worth** | 0.50 (0.32) | 0.63 (0.27) |
|  | **Blaming yourself** | 0.52 (0.31) | 0.70 (0.26) |
|  | **Difficulties making decisions** | 0.38 (0.29) | 0.53 (0.31) |
|  | **Functional impairment (relationships)** | 0.40 (0.30) | 0.53 (0.31) |
|  | **Short-tempered** | 0.43 (0.28) | 0.64 (0.31) |
|  | **Functional impairment (work)** | 0.32 (0.29) | 0.60 (0.35) |
|  | **Unable to relax** | 0.45 (0.26) | 0.61 (0.27) |
|  | **Harder to concentrate** | 0.39 (0.29) | 0.66 (0.30) |
|  | **Functional impairment (leisure)** | 0.35 (0.31) | 0.61 (0.29) |
|  | **Reduced sex drive** | 0.37 (0.35) | 0.55 (0.38) |
|  | **Slowed down mentally/physically** | 0.24 (0.24) | 0.36 (0.27) |
|  | **Tiredness** | 0.49 (0.29) | 0.81 (0.24) |
|  | **Leaden paralysis** | 0.47 (0.30) | 0.80 (0.23) |
|  | **Interpersonal rejection sensitivity** | 0.52 (0.31) | 0.69 (0.30) |
|  | **Diurnal mood variation** | 0.21 (0.26) | 0.17 (0.27) |
|  | **Decreased interest** | 0.39 (0.32) | 0.61 (0.30) |
|  | **Decreased enjoyment** | 0.34 (0.26) | 0.54 (0.22) |
|  | **Irritability** | 0.41 (0.29) | 0.59 (0.27) |
|  | **Significant weight change** | 0.57 (0.23) | 0.45 (0.28) |
|  | **Hypersomnia** | 0.25 (0.32) | 0.27 (0.31) |
|  | **Psychomotor retardation** | 0.20 (0.23) | 0.34 (0.26) |
|  | **Restlessness** | 0.19 (0.24) | 0.41 (0.28) |
|  | **Easily fatigued** | 0.47 (0.26) | 0.67 (0.23) |
|  | **Feelings of worthlessness** | 0.56 (0.29) | 0.73 (0.23) |
|  | **Excessive or inappropriate guilt** | 0.41 (0.33) | 0.63 (0.27) |
|  | **Difficulty concentrating** | 0.45 (0.25) | 0.59 (0.24) |
|  | **Indecisiveness** | 0.38 (0.28) | 0.54 (0.31) |
| **Generalized anxiety** | **Excessive worrying** | 0.25 (0.39) | 0.61 (0.44) |
|  | **Unwanted thoughts** | 0.22 (0.36) | 0.60 (0.44) |
|  | **Restless and unable to relax** | 0.16 (0.28) | 0.48 (0.38) |
|  | **Muscle tension** | 0.18 (0.31) | 0.52 (0.39) |
|  | **Problems sleeping** | 0.18 (0.31) | 0.49 (0.41) |
|  | **Energy levels** | 0.14 (0.25) | 0.47 (0.36) |
|  | **Concentration problems** | 0.11 (0.21) | 0.41 (0.35) |
|  | **Easily annoyed or irritated** | 0.14 (0.27) | 0.48 (0.39) |
|  | **Fidgety** | 0.07 (0.19) | 0.22 (0.26) |
|  | **Impairment in functioning** | 0.12 (0.23) | 0.38 (0.32) |
|  | **Tired more easily than usual** | 0.19 (0.34) | 0.58 (0.42) |
|  | **Emotional distress** | 0.21 (0.35) | 0.59 (0.43) |
| **Bipolar/mania** | **Inflated self-esteem/grandiosity** | 0.36 (0.27) | 0.35 (0.32) |
|  | **More active** | 0.34 (0.25) | 0.43 (0.28) |
|  | **Sociability** | 0.37 (0.29) | 0.38 (0.27) |
|  | **Decreased need for sleep** | 0.23 (0.28) | 0.24 (0.27) |
|  | **Functional impairment (work)** | 0.09 (0.19) | 0.14 (0.26) |
|  | **More talkative** | 0.22 (0.24) | 0.29 (0.28) |
|  | **Functional impairment (relationships)** | 0.11 (0.23) | 0.20 (0.29) |
|  | **Racing thoughts** | 0.36 (0.29) | 0.42 (0.29) |
|  | **More talkative (others)** | 0.20 (0.40) | 0.34 (0.48) |
|  | **Everywhere/everything** | 0.32 (0.28) | 0.35 (0.33) |
|  | **Psychomotor agitation** | 0.28 (0.25) | 0.43 (0.32) |
|  | **Loss of social inhibition** | 0.21 (0.27) | 0.26 (0.28) |
|  | **Recklessness** | 0.36 (0.48) | 0.48 (0.50) |
|  | **Functional impairment (leisure)** | 0.09 (0.18 | 0.14 (0.25) |
|  | **Difficulty concentrating** | 0.32 (0.26) | 0.39 (0.26) |
|  | **Creativity** | 0.33 (0.29) | 0.41 (0.32) |
|  | **Delusions** | 0.13 (0.34) | 0.13 (0.33) |
|  | **Hallucinations** | 0.06 (0.25) | 0.25 (0.37) |
|  | **Mood incongruency** | 0.04 (0.19) | 0.08 (0.27) |
| **Hypomania** | **Motivation** | 0.22 (0.25) | 0.29 (0.34) |
|  | **More talkative** | 0.18 (0.25) | 0.23 (0.29) |
|  | **Meeting new people** | 0.17 (0.24) | 0.24 (0.23) |
|  | **Inflated self-esteem/grandiosity** | 0.11 (0.20) | 0.20 (0.22) |
|  | **Increased energy** | 0.11 (0.20) | 0.21 (0.29) |
|  | **Life/soul of the party** | 0.10 (0.19) | 0.11 (0.23) |
|  | **Sexual feelings/thoughts** | 0.19 (0.28) | 0.23 (0.31) |
|  | **Heightened senses** | 0.04 (0.11) | 0.10 (0.22) |
|  | **Creativity** | 0.06 (0.14) | 0.13 (0.24) |
|  | **Psychotic delusions** | 0.07 (0.17) | 0.17 (0.27) |
|  | **Flirtatious/sexual** | 0.16 (0.25) | 0.22 (0.32) |
|  | **Productivity** | 0.19 (0.26) | 0.31 (0.39) |
|  | **Recklessness** | 0.19 (0.27) | 0.31 (0.35) |
|  | **Jokes/puns** | 0.19 (0.27) | 0.23 (0.33) |
|  | **Racing thoughts** | 0.14 (0.23) | 0.23 (0.33) |
| **Social anxiety** | **Worried about showing anxiety symptoms** | 0.40 (0.36) | 0.51 (0.40) |
|  | **Social/performance situations** | 0.33 (0.34) | 0.41 (0.36) |
|  | **Avoidance of social situations** | 0.29 (0.32) | 0.44 (0.40) |
|  | **Out of proportion** | 0.30 (0.35) | 0.38 (0.36) |
|  | **Duration of social anxiety problems** | 0.46 (0.48) | 0.65 (0.48) |
|  | **Functional impairment** | 0.25 (0.32) | 0.32 (0.36) |
| **Emotional instability** | **Mood lability** | 0.47 (0.37) | 0.70 (0.33) |
|  | **Fear of abandonment** | 0.38 (0.40) | 0.62 (0.41) |
|  | **Relationship issues** | 0.16 (0.29) | 0.23 (0.30) |
|  | **Anger issues** | 0.26 (0.32) | 0.40 (0.35) |
|  | **Self-image instability** | 0.27 (0.29) | 0.38 (0.32) |
|  | **Self-esteem** | 0.44 (0.40) | 0.78 (0.32) |
|  | **Recklessness** | 0.20 (0.30) | 0.31 (0.35 |
|  | **Feeling empty/lonely** | 0.38 (0.37) | 0.71 (0.32) |
|  | **Duration of emotional instability problems** | 0.43 (0.39) | 0.61 (0.32) |
|  | **Trait-like symptoms** | 0.46 (0.50) | 0.44 (0.50) |
|  | **Self-harm** | 0.19 (0.26) | 0.38 (0.23) |
| **Panic disorder** | **Unexpected panic attacks** | 0.54 (0.50) | 0.80 (0.41) |
|  | **Frequency of panic attacks** | 0.32 (0.39) | 0.67 (0.41) |
|  | **Pounding heart** | 0.41 (0.49) | 0.78 (0.42) |
|  | **Excessive sweating** | 0.28 (0.45) | 0.56 (0.50) |
|  | **Trembling** | 0.31 (0.47) | 0.56 (0.50) |
|  | **Shortness of breath** | 0.34 (0.47) | 0.63 (0.49) |
|  | **Choking** | 0.06 (0.23) | 0.09 (0.29) |
|  | **Chest pain** | 0.18 (0.38) | 0.34 (0.48) |
|  | **Nausea** | 0.24 (0.43) | 0.47 (0.50) |
|  | **Dizziness** | 0.32 (0.47) | 0.63 (0.49) |
|  | **Fear of dying** | 0.09 (0.29) | 0.09 (0.29) |
|  | **Fear of losing control** | 0.27 (0.45) | 0.47 (0.50) |
|  | **Worried about additional attacks** | 0.18 (0.38) | 0.38 (0.48) |
|  | **Change in behavior** | 0.15 (0.36) | 0.44 (0.50) |
| **Obsessive-compulsive disorder** | **Obsessions** | 0.40 (0.49) | 0.63 (0.49) |
|  | **Compulsions** | 0.24 (0.43) | 0.39 (0.49) |
|  | **Distress** | 0.14 (0.23) | 0.34 (0.34) |
|  | **Attempts to ignore/suppress** | 0.28 (0.45) | 0.55 (0.50) |
|  | **Recognition** | 0.37 (0.48) | 0.56 (0.50) |
|  | **Inappropriate/excessive** | 0.11 (0.22) | 0.21 (0.30) |
|  | **Functional impairment** | 0.04 (0.14) | 0.14 (0.28) |
|  | **Duration per day** | 0.06 (0.18) | 0.14 (0.27) |
|  | **Duration of obsessive-compulsive problems** | 0.24 (0.41) | 0.50 (0.48) |
| **Eating disorder** | **Fear of eating/being overweight** | 0.33 (0.47) | 0.34 (0.48) |
|  | **Diagnosed/belief** | 0.10 (0.30) | 0.17 (0.38) |
| **Insomnia** | **Sleep problems** | 0.40 (0.28) | 0.64 (0.30) |
|  | **Sleep satisfaction** | 0.36 (0.30) | 0.64 (0.33) |
|  | **Time taken to fall asleep** | 0.35 (0.35) | 0.53 (0.37) |
|  | **Restless/unsatisfying sleep** | 0.41 (0.39) | 0.69 (0.39) |
|  | **Broken/unsatisfying sleep** | 0.30 (0.31) | 0.59 (0.33) |
|  | **Waking up early** | 0.22 (0.30) | 0.37 (0.37) |
|  | **Frequency of sleep problems** | 0.29 (0.35) | 0.60 (0.38) |
|  | **Duration of sleep problems** | 0.54 (0.46) | 0.71 (0.40) |
|  | **Functional impairment** | 0.22 (0.26) | 0.48 (0.31) |
|  | **Distress** | 0.15 (0.23) | 0.38 (0.34) |

**Note.** While all participants were asked about symptoms of depression, BD/mania, and hypomania, the questions for the remaining conditions were adaptive in nature, such that only relevant questions were asked based on responses to previous questions. This resulted in 13.44% of subthreshold depression and 6.67% of MDD responses being ‘not applicable’. These responses were imputed as zeros. Furthermore, due to the adaptive nature of the digital mental health assessment, participants answered questions on current (i.e., present in the last two weeks) *or* past symptoms of all disorders. Scores per symptom ranged from zero to one, with higher scores indicating increased severity.
