# Supplementary material for: Toward an Extended Definition of Major Depressive Disorder Symptomatology: Digital Assessment and Cross-validation Study
Source: JMIR Form Res. 2021 Oct 28;5(10):e27908. doi: 10.2196/27908 (PMC8587324; doi:10.2196/27908)
Supplement: Multimedia Appendix 2 [file formative_v5i10e27908_app2.docx]

***Multimedia Appendix 2*Table 2.** Depression model: mean relative feature importances

| **Feature** | **Mean Relative Importance** |
| --- | --- |
| Leaden paralysis | 0.135 |
| Tiredness | 0.111 |
| Low energy | 0.079 |
| Harder to concentrate | 0.076 |
| Functional impairment (work) | 0.060 |
| Restlessness | 0.053 |
| Functional impairment (leisure) | 0.039 |
| Excessive or inappropriate guilt | 0.038 |
| Short-tempered | 0.036 |
| Easily annoyed | 0.033 |
| Easily fatigued | 0.030 |
| Functional impairment (home) | 0.030 |
| Decreased enjoyment | 0.027 |
| Irritability | 0.027 |
| Blaming yourself | 0.022 |
| Significant weight change | 0.021 |
| Functional impairment (relationships) | 0.020 |
| Decreased interest | 0.018 |
| Large appetite | 0.017 |
| Unable to relax | 0.015 |
| Interpersonal rejection sensitivity | 0.015 |
| Psychomotor retardation | 0.014 |
| Feelings of worthlessness | 0.014 |
| Reduced sex drive | 0.012 |
| Indecisiveness | 0.009 |
| Difficulty concentrating | 0.008 |
| Difficulties making decisions | 0.008 |
| Slowed down mentally/physically | 0.007 |
| Low self-worth | 0.007 |
| Small appetite | 0.006 |
| Hypersomnia | 0.005 |
| Diurnal mood variation | 0.004 |
| Sleeping too much | 0.003 |
| Struggle to fall asleep | 0.002 |
| Unsatisfying sleep | 0.001 |
| Waking up early | 0.001 |
