# Supplementary material for: Toward an Extended Definition of Major Depressive Disorder Symptomatology: Digital Assessment and Cross-validation Study
Source: JMIR Form Res. 2021 Oct 28;5(10):e27908. doi: 10.2196/27908 (PMC8587324; doi:10.2196/27908)
Supplement: Multimedia Appendix 3 [file formative_v5i10e27908_app3.docx]

***Multimedia Appendix 3***

**Table 3.** Depression model: percentage feature occurrences

| **Feature** | **Percentage Occurrence** |
| --- | --- |
| Leaden paralysis | 99.67 |
| Tiredness | 98.33 |
| Harder to concentrate | 96.67 |
| Restlessness | 95.67 |
| Low energy | 95.67 |
| Functional impairment (work) | 94.00 |
| Excessive or inappropriate guilt | 93.67 |
| Easily annoyed | 93.00 |
| Functional impairment (leisure) | 91.33 |
| Functional impairment (home) | 91.00 |
| Short-tempered | 90.67 |
| Decreased enjoyment | 89.33 |
| Irritability | 88.33 |
| Easily fatigued | 88.00 |
| Functional impairment (relationships) | 87.67 |
| Blaming yourself | 87.00 |
| Significant weight change | 85.33 |
| Unable to relax | 84.67 |
| Decreased interest | 84.67 |
| Large appetite | 83.00 |
| Interpersonal rejection sensitivity | 82.33 |
| Feelings of worthlessness | 82.00 |
| Psychomotor retardation | 81.33 |
| Reduced sex drive | 77.67 |
| Indecisiveness | 77.33 |
| Difficulty concentrating | 75.33 |
| Difficulties making decisions | 74.33 |
| Slowed down mentally/physically | 72.33 |
| Low self-worth | 72.00 |
| Hypersomnia | 60.67 |
| Diurnal mood variation | 60.67 |
| Small appetite | 59.67 |
| Sleeping too much | 56.67 |
| Unsatisfying sleep | 45.67 |
| Waking up early | 44.00 |
| Struggle to fall asleep | 43.67 |
