# Supplementary material for: Toward an Extended Definition of Major Depressive Disorder Symptomatology: Digital Assessment and Cross-validation Study
Source: JMIR Form Res. 2021 Oct 28;5(10):e27908. doi: 10.2196/27908 (PMC8587324; doi:10.2196/27908)
Supplement: Multimedia Appendix 4 [file formative_v5i10e27908_app4.docx]

***Multimedia Appendix 4***


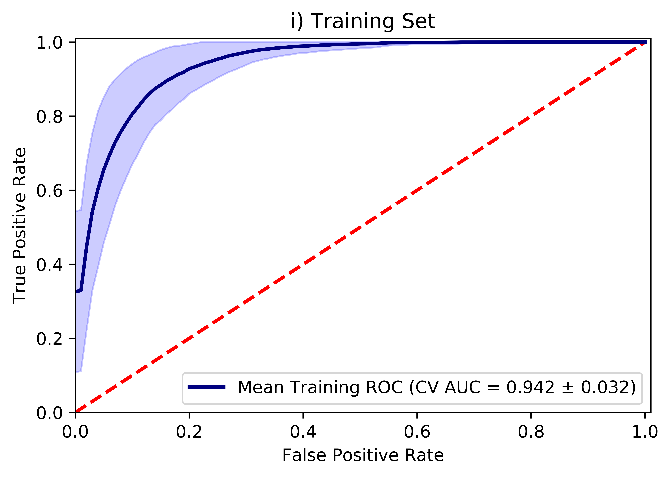

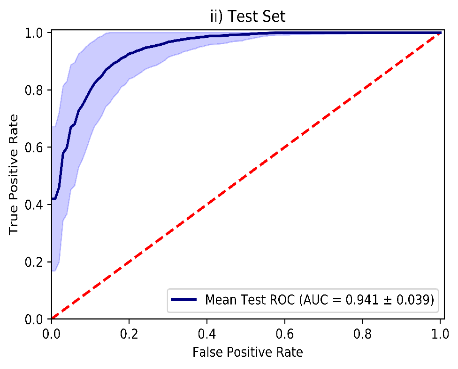


**Figure 1. ROC curves showing mean predictive performance of the extended model.** The models were applied to predict the probability of MDD in the: (i) training and (ii) test sets.

*Key:* AUC, area under the curve; CV AUC, cross-validated area under the curve; MDD, Major Depressive Disorder; ROC, receiver operating characteristic.
