# Supplementary material for: Toward an Extended Definition of Major Depressive Disorder Symptomatology: Digital Assessment and Cross-validation Study
Source: JMIR Form Res. 2021 Oct 28;5(10):e27908. doi: 10.2196/27908 (PMC8587324; doi:10.2196/27908)
Supplement: Multimedia Appendix 5 [file formative_v5i10e27908_app5.docx]

***Multimedia Appendix 5***

**Table 4.** Extended model: mean relative feature importances colored by disorder/symptom cluster

| **Feature** | **Mean Relative Importance** |
| --- | --- |
| Leaden paralysis | 0.050 |
| Tiredness | 0.043 |
| Low energy | 0.032 |
| Harder to concentrate | 0.027 |
| Easily annoyed or irritated | 0.026 |
| Tired more easily than usual | 0.025 |
| Energy levels | 0.024 |
| Functional impairment (work) | 0.023 |
| Concentration problems | 0.023 |
| Emotional distress | 0.022 |
| Frequency of sleep problems | 0.022 |
| Restless and unable to relax | 0.021 |
| Feeling empty/lonely | 0.021 |
| Self-esteem | 0.021 |
| Unwanted thoughts | 0.019 |
| Impairment in functioning | 0.019 |
| Frequency of panic attacks | 0.019 |
| Restlessness | 0.018 |
| Sleep problems | 0.018 |
| Muscle tension | 0.017 |
| Broken/unsatisfying sleep | 0.017 |
| Functional impairment | 0.017 |
| Distress | 0.016 |
| Self-harm | 0.015 |
| Functional impairment (leisure) | 0.014 |
| Excessive or inappropriate guilt | 0.013 |
| Excessive worrying | 0.013 |
| Problems sleeping | 0.013 |
| Sleep satisfaction | 0.012 |
| Short-tempered | 0.012 |
| Easily annoyed | 0.012 |
| Functional impairment (home) | 0.011 |
| Pounding heart | 0.011 |
| Fidgety | 0.011 |
| Easily fatigued | 0.011 |
| Decreased enjoyment | 0.010 |
| Restless/unsatisfying sleep | 0.010 |
| Significant weight change | 0.008 |
| Irritability | 0.008 |
| Distress | 0.008 |
| Blaming yourself | 0.007 |
| Functional impairment (relationships) | 0.007 |
| Unable to relax | 0.007 |
| Decreased interest | 0.006 |
| Feelings of worthlessness | 0.006 |
| Duration of obsessive-compulsive symptoms | 0.006 |
| Interpersonal rejection sensitivity | 0.006 |
| Psychomotor agitation | 0.006 |
| Mood lability | 0.006 |
| Change in behaviour | 0.006 |
| Large appetite | 0.005 |
| Dizziness | 0.005 |
| Functional impairment | 0.005 |
| Waking up early | 0.005 |
| Reduced sex drive | 0.005 |
| Excessive sweating | 0.005 |
| Productivity | 0.005 |
| Fear or abandonment | 0.005 |
| Time taken to fall asleep | 0.005 |
| Psychomotor retardation | 0.004 |
| Attempts to ignore/suppress | 0.004 |
| Difficulties making decisions | 0.004 |
| Shortness of breath | 0.004 |
| Duration of emotional instability | 0.004 |
| Indecisiveness | 0.004 |
| Creativity | 0.004 |
| More active | 0.003 |
| Motivation | 0.003 |
| Slowed down mentally/physically | 0.003 |
| Anger issues | 0.003 |
| Trembling | 0.003 |
| Difficulty concentrating | 0.003 |
| Recklessness | 0.003 |
| Sociability | 0.003 |
| Recklessness | 0.003 |
| Increased energy | 0.003 |
| Low self-worth | 0.003 |
| Racing thoughts | 0.003 |
| Avoidance of social situations | 0.003 |
| Duration of sleep problems | 0.003 |
| Difficulty concentrating | 0.003 |
| Decreased need for sleep | 0.003 |
| Unexpected panic attacks | 0.003 |
| Inflated self-esteem/grandiosity | 0.003 |
| Self-image instability | 0.003 |
| Relationship issues | 0.003 |
| Worried about showing anxiety symptoms | 0.003 |
| Psychotic delusions | 0.002 |
| Inappropriate/excessive | 0.002 |
| Functional impairment (relationships) | 0.002 |
| Social/performance situations | 0.002 |
| Small appetite | 0.002 |
| Duration of social anxiety | 0.002 |
| Hypersomnia | 0.002 |
| Everywhere/everything | 0.002 |
| Sexual feelings/thoughts | 0.002 |
| Nausea | 0.002 |
| Loss of social inhibition | 0.002 |
| Functional impairment (work) | 0.002 |
| Racing thoughts | 0.002 |
| Out of proportion | 0.002 |
| More talkative | 0.002 |
| Flirtatious/sexual | 0.002 |
| Diurnal mood variation | 0.002 |
| Obsessions | 0.002 |
| Chest pain | 0.002 |
| More talkative | 0.002 |
| Creativity | 0.002 |
| Sleeping too much | 0.002 |
| Meeting new people | 0.002 |
| Functional impairment | 0.002 |
| Jokes/puns | 0.002 |
| Functional impairment (leisure) | 0.002 |
| Fear of losing control | 0.002 |
| Duration per day | 0.002 |
| Life/soul of the party | 0.002 |
| Fear of dying | 0.001 |
| Recklessness | 0.001 |
| Heightened senses | 0.001 |
| Worried about additional attacks | 0.001 |
| Recognition | 0.001 |
| Inflated self-esteem/grandiosity | 0.001 |
| Hallucinations | 0.001 |
| Fear of eating/being overweight | 0.001 |
| Diagnosed/belief | 0.001 |
| Trait-like symptoms | 0.001 |
| Compulsions | 0.001 |
| More talkative (others) | 0.001 |
| Waking up early | 0.001 |
| Delusions | 0.001 |
| Choking | 0.001 |
| Mood incongruency | 0.001 |
| Struggle to fall asleep | 0.001 |
| Unsatisfying sleep | 0.001 |

***Note.*** Depression; Insomnia; Generalized anxiety disorder; Emotional instability;
 Panic disorder; Social anxiety; Bipolar disorder; Hypomania; Obsessive-compulsive disorder;
 Eating disorders
