# Supplementary material for: Toward an Extended Definition of Major Depressive Disorder Symptomatology: Digital Assessment and Cross-validation Study
Source: JMIR Form Res. 2021 Oct 28;5(10):e27908. doi: 10.2196/27908 (PMC8587324; doi:10.2196/27908)
Supplement: Multimedia Appendix 6 [file formative_v5i10e27908_app6.docx]

***Multimedia Appendix 6***

**Table 5.** Extended model: percentage feature occurrences colored by disorder/symptom cluster

| **Feature** | **Percentage Occurrence** |
| --- | --- |
| Tiredness | 99.67 |
| Leaden paralysis | 98.00 |
| Easily annoyed or irritated | 96.67 |
| Tired more easily than usual | 96.33 |
| Low energy | 96.33 |
| Emotional distress | 96.00 |
| Restless and unable to relax | 95.67 |
| Harder to concentrate | 95.67 |
| Concentration problems | 95.00 |
| Energy levels | 94.67 |
| Impairment in functioning | 94.33 |
| Distress | 94.00 |
| Sleep problems | 94.00 |
| Frequency of panic attacks | 94.00 |
| Feeling empty/lonely | 94.00 |
| Restlessness | 93.67 |
| Broken/unsatisfying sleep | 93.67 |
| Self-esteem | 93.67 |
| Unwanted thoughts | 93.00 |
| Functional impairment (work) | 93.00 |
| Frequency of sleep problems | 93.00 |
| Muscle tension | 92.33 |
| Excessive or inappropriate guilt | 92.33 |
| Functional impairment (leisure) | 92.33 |
| Self-harm | 92.33 |
| Decreased enjoyment | 92.00 |
| Functional impairment (home) | 91.67 |
| Short-tempered | 91.00 |
| Functional impairment | 91.00 |
| Excessive worrying | 90.67 |
| Problems sleeping | 90.67 |
| Easily annoyed | 90.33 |
| Sleep satisfaction | 90.33 |
| Pounding heart | 89.33 |
| Fidgety | 88.33 |
| Restless/unsatisfying sleep | 88.00 |
| Easily fatigued | 87.00 |
| Distress | 87.00 |
| Irritability | 86.67 |
| Decreased interest | 86.33 |
| Functional impairment (relationships) | 86.33 |
| Significant weight change | 85.67 |
| Blaming yourself | 85.00 |
| Interpersonal rejection sensitivity | 84.00 |
| Unable to relax | 83.67 |
| Duration of obsessive-compulsive symptoms | 83.67 |
| Functional impairment | 83.33 |
| Difficulties making decisions | 82.67 |
| Feelings of worthlessness | 82.33 |
| Mood lability | 82.33 |
| Reduced sex drive | 82.00 |
| Psychomotor agitation | 81.67 |
| Difficulty concentrating | 81.33 |
| Large appetite | 81.33 |
| Indecisiveness | 81.00 |
| Dizziness | 81.00 |
| Change in behaviour | 80.67 |
| Time taken to fall asleep | 80.33 |
| Duration of emotional instability | 80.33 |
| Psychomotor retardation | 80.00 |
| Excessive sweating | 80.00 |
| Fear or abandonment | 80.00 |
| Waking up early | 79.33 |
| Creativity | 78.33 |
| Productivity | 78.33 |
| Shortness of breath | 78.33 |
| Low self-worth | 78.00 |
| Attempts to ignore/suppress | 78.00 |
| Anger issues | 77.67 |
| More active | 76.33 |
| Slowed down mentally/physically | 76.33 |
| Recklessness | 76.33 |
| Recklessness | 75.33 |
| Trembling | 75.00 |
| Decreased need for sleep | 74.33 |
| Racing thoughts | 74.33 |
| Increased energy | 74.33 |
| Inappropriate/excessive | 73.67 |
| Relationship issues | 73.67 |
| Difficulty concentrating | 73.00 |
| Motivation | 73.00 |
| Psychotic delusions | 73.00 |
| Functional impairment (relationships) | 72.67 |
| Sociability | 72.67 |
| Self-image instability | 72.67 |
| Everywhere/everything | 72.33 |
| Duration of sleep problems | 72.00 |
| Avoidance of social situations | 72.00 |
| Duration of social anxiety | 72.00 |
| Inflated self-esteem/grandiosity | 71.67 |
| Functional impairment (work) | 71.33 |
| Racing thoughts | 71.33 |
| Unexpected panic attacks | 71.33 |
| Hypersomnia | 71.00 |
| Small appetite | 71.00 |
| Chest pain | 71.00 |
| Worried about showing anxiety symptoms | 71.00 |
| Diurnal mood variation | 70.67 |
| More talkative | 70.67 |
| Nausea | 70.67 |
| Loss of social inhibition | 70.33 |
| Creativity | 70.33 |
| Social/performance situations | 70.00 |
| Sexual feelings/thoughts | 69.33 |
| More talkative | 69.00 |
| Functional impairment | 69.00 |
| Out of proportion | 69.00 |
| Flirtatious/sexual | 68.33 |
| Sleeping too much | 67.67 |
| Jokes/puns | 67.33 |
| Heightened senses | 67.00 |
| Meeting new people | 67.00 |
| Functional impairment (leisure) | 66.67 |
| Fear of dying | 66.67 |
| Life/soul of the party | 66.33 |
| Recklessness | 66.00 |
| Fear of losing control | 66.00 |
| Duration per day | 65.33 |
| Obsessions | 65.00 |
| Worried about additional attacks | 64.33 |
| Inflated self-esteem/grandiosity | 63.00 |
| Trait-like symptoms | 62.33 |
| Recognition | 62.00 |
| Fear of eating/being overweight | 60.00 |
| Waking up early | 59.67 |
| Diagnosed/belief | 59.67 |
| Compulsions | 59.67 |
| More talkative (others) | 59.33 |
| Hallucinations | 58.00 |
| Delusions | 57.00 |
| Choking | 56.33 |
| Mood incongruency | 56.00 |
| Unsatisfying sleep | 52.67 |
| Struggle to fall asleep | 52.33 |

***Note.*** Depression; Insomnia; Generalized anxiety disorder; Emotional instability;
 Panic disorder; Social anxiety; Bipolar disorder; Hypomania; Obsessive-compulsive disorder;
 Eating disorders
