# Supplementary material for: Toward an Extended Definition of Major Depressive Disorder Symptomatology: Digital Assessment and Cross-validation Study
Source: JMIR Form Res. 2021 Oct 28;5(10):e27908. doi: 10.2196/27908 (PMC8587324; doi:10.2196/27908)
Supplement: Multimedia Appendix 7 [file formative_v5i10e27908_app7.docx]

***Multimedia Appendix 7***

**Table 6.** Truncated model: percentage feature occurrences colored by disorder/symptom cluster

| **Feature** | **Percentage Occurrence** |
| --- | --- |
| Tiredness | 99.67 |
| Functional impairment (work) | 99.33 |
| Frequency of sleep problems | 99.33 |
| Tired more easily than usual | 99.00 |
| Leaden paralysis | 98.67 |
| Easily annoyed or irritated | 98.33 |
| Restless and unable to relax | 98.33 |
| Harder to concentrate | 98.33 |
| Energy levels | 98.00 |
| Self-esteem | 98.00 |
| Concentration problems | 97.67 |
| Emotional distress | 97.67 |
| Impairment in functioning | 97.67 |
| Low energy | 97.67 |
| Distress | 97.67 |
| Functional impairment | 97.67 |
| Sleep problems | 97.67 |
| Unwanted thoughts | 97.33 |
| Easily annoyed | 97.33 |
| Functional impairment (leisure) | 97.33 |
| Restlessness | 97.33 |
| Broken/unsatisfying sleep | 97.33 |
| Feeling empty/lonely | 97.33 |
| Frequency of panic attacks | 97.00 |
| Problems sleeping | 96.67 |
| Sleep satisfaction | 96.67 |
| Self-harm | 96.67 |
| Decreased enjoyment | 96.33 |
| Short-tempered | 96.33 |
| Muscle tension | 95.67 |
| Excessive or inappropriate guilt | 95.67 |
| Functional impairment (home) | 95.33 |
| Excessive worrying | 94.67 |

***Note.*** Depression; Insomnia; Generalized anxiety disorder; Emotional instability;
 Panic disorder
